# Antibacterial Effects of Black Cumin Seed Oil on Oral Microcosm Biofilms

**DOI:** 10.3390/microorganisms12102098

**Published:** 2024-10-20

**Authors:** Ahyun Jo, Hee-Eun Kim

**Affiliations:** 1 Department of Health Science, Gachon University Graduate School of Public Health, Incheon 21936, Republic of Korea; ahyun990720@gmail.com; 2 Department of Dental Hygiene, Gachon University College of Medical Science, Incheon 21936, Republic of Korea

**Keywords:** antibacterial, oral microcosm biofilm, black cumin seed oil, chlorhexidine, extracellular polysaccharide, colony-forming units

## Abstract

Interest in natural extracts for managing oral biofilms is increasing, with black cumin seed oil (BCSO) demonstrating efficacy against *Streptococcus mutans*. The effectiveness of antibacterial agents should be evaluated using multi-species oral biofilm models that closely mimic actual conditions. This study aimed to compare the antibacterial effects of BCSO and chlorhexidine gluconate (CHX) on oral microcosm biofilms. Biofilms using human saliva as the inoculum were cultured for 2 days and subsequently treated with 0.5% dimethyl sulfoxide, 0.5% BCSO, or 0.12% CHX once daily for 6 days. Following treatment, the red fluorescence intensity (Ratio_R/G_) of the oral biofilm; biomass, including extracellular polymeric substance (EPS) levels and live bacteria counts; and colony-forming units (CFUs) of aciduric bacteria were evaluated. Ratio_R/G_ after BCSO treatment (1.26 ± 0.03) was not significantly different from that after CHX treatment (*p* = 0.552). The EPS levels were also not significantly different between the two groups (*p* = 0.743). The live bacteria count was 0.55 times lower in the BCSO-treated group than in the CHX-treated group (*p* = 0.018). No significant between-group difference was observed in the CFUs of aciduric bacteria (*p* = 0.935). These results suggest that BCSO exhibits antibacterial effects similar to those of CHX, highlighting its potential as an effective alternative.

## 1. Introduction

Oral biofilms are structured microbial communities within a three-dimensional extracellular polymeric substance (EPS) [1]. They are recognized as primary etiological factors for dental caries and periodontal diseases [2,3]. A diverse array of microorganisms coexist within oral biofilms, maintaining a stable equilibrium through dynamic interactions among resident species [4]. These interactions, including synergies and antagonisms, maintain a stable microbial composition despite minor environmental stressors [5]. Environmental factors such as frequent sugar intake, poor oral hygiene, and xerostomia can disrupt this microbial balance, leading to an ecological shift that favors aciduric and acidogenic bacteria associated with dental caries. Consequently, the pathogenicity of the oral biofilm increases, ultimately resulting in the onset of oral diseases [6].

Effective management of oral biofilms involves both mechanical and chemical control [7]. Chlorhexidine gluconate (CHX) is currently regarded as the most potent chemotherapeutic agent and the gold standard for oral biofilm management. CHX, a cationic compound, binds to negatively charged bacterial cell walls, destroying cell membranes and causing cell lysis [8]. However, prolonged CHX use can cause side effects such as tooth discoloration, xerostomia, altered taste, oral burning, mucosal irritation, parotid gland swelling, and most concerningly, antibacterial resistance [9]. In addition, CHX’s non-targeted action may not address the microbial imbalance and could further disrupt the symbiotic community [10].

Recently, interest in natural extracts with fewer side effects has grown [11,12,13]. The World Health Organization (WHO) reported that 60–80% of the global population residing in developing countries relies on herbal remedies for primary healthcare and recommends using medicinal plants to develop effective healthcare programs [14]. Among various natural substances, black cumin seed, derived from the seeds of the *Nigella sativa (N. sativa)* plant native to the Mediterranean region, has attracted significant attention. Previous studies have reported that black cumin seed oil (BCSO) and extracts exhibit potent antibacterial effects against both Gram-positive and Gram-negative bacteria, including resistant strains [15,16]. While BCSO’s efficacy in systemic diseases is well documented [17,18], its effects on oral diseases have not been thoroughly investigated. A few previous studies have shown that BCSO not only shows antibacterial effects against *Streptococcus mutans*, the primary producer of EPS for the formation of oral biofilms, but also inhibits the adhesion of *S. mutans* to tooth surfaces [19,20]. However, these studies evaluated the effects of BCSO only on single bacterial species. 

Oral diseases result from interactions among hundreds of microorganisms, not a single species [20,21]. Given the promising results of BCSO against single bacteria species, BCSO might exert similar effects in a multi-species environment, reflecting the diverse interactions among microorganisms in the oral cavity. Therefore, to develop effective strategies for managing oral diseases, testing antibacterial agents using multi-species oral biofilm models that closely resemble actual oral biofilms is essential [22]. The oral microcosm biofilm model is recommended as the most accurate in vitro model to replicate the actual oral environment [23]. This model uses human saliva as an inoculum, which allows it to simulate the interactions among diverse bacterial species, oral biofilm formation, and acid production, closely mirroring actual oral conditions [24]. Accordingly, to enhance the clinical relevance of our findings, we evaluated the antibacterial effects of BCSO using the oral microcosm biofilm model in the present study. This study aimed to compare the antibacterial effects of BCSO with those of CHX on oral microcosm biofilms. Our null hypothesis was that there would be no significant difference between the antibacterial effects of BCSO and those of CHX on oral microcosm biofilms. 

## 2. Materials and Methods

### 2.1. Ethical Considerations and Criteria for Participants

This study was approved by the Institutional Review Board of Gachon University (Approval No. 1044396-202312-HR-237-01). A healthy adult woman with no active dental caries or periodontal disease and no history of antibiotic use in the past 3 months was recruited for saliva collection. The participant was thoroughly informed about the purpose and methods of the study, and saliva samples were collected after obtaining written informed consent.

### 2.2. Sample Size Determination and Specimen Preparation

The required sample size for the experiment was calculated using G*Power ver. 3.1.9.7 (Heinrich-Heine-University Düsseldorf, Düsseldorf, Germany). Based on preliminary experimental results, with an effect size of 0.43, a significance level of 0.05, and a power of 0.80, a minimum of 57 samples were needed for one-way analysis of variance (ANOVA).

**Figure 1:**
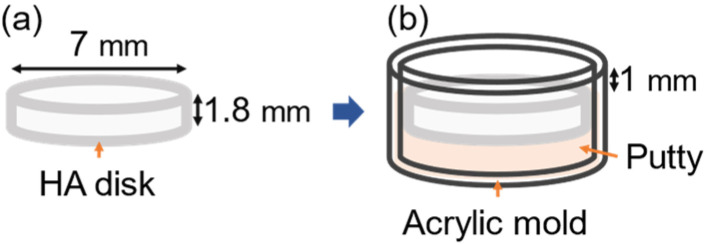
Specimen preparation. (**a**) HA disk, (**b**) HA disk embedded in an acrylic mold, leaving a 1 mm space above the disk for oral microcosm biofilm formation. HA, hydroxyapatite.

To create the oral microcosm biofilm, we prepared hydroxyapatite (HA) disks (Himed, Old Bethpage, NY, USA) with a diameter of 7 mm and height of 1.8 mm as substrates. These HA disks were placed in a circular acrylic mold using dental putty impression material (AFFINIS putty soft, Coltene, Altstä tten, Switzerland). The HA disks were positioned at the bottom of the acrylic mold, with a 1 mm space left above the disks for oral biofilm formation (Figure 1).

### 2.3. Preparation of 0.5% BCSO

To prepare the 0.5% BCSO solution, 0.15 mL of cold-pressed pure BCSO (PREMIUM Black Seed Oil, Amazing herbs, Buford, GA, USA) was mixed with 29.85 mL of 0.5% dimethyl sulfoxide (DMSO), which served as the organic solvent. To ensure uniform mixing, the solution was sonicated using an ultrasonic vibrator (SHB-1025; Saehan Sonic, Seoul, Republic of Korea) for 5 min, followed by vortexing with a vortex mixer (VM-96A; Lab companion, Seoul, Republic of Korea) at 3000 revolutions per min for 5 min.

### 2.4. Formation of the Oral Microcosm Biofilm

A saliva donor was instructed to refrain from any oral hygiene activities for 24 h before saliva collection. Stimulated saliva was collected by having the participant chew flavorless, odorless paraffin wax, which yielded 25 mL of saliva. The collected saliva was filtered through sterilized glass wool (Kanto Chemical, Tokyo, Japan) to remove particulate matter. Each specimen was inoculated with 1.5 mL of saliva and incubated at 37 °C with 10% carbon dioxide (CO_2)_ for 4 h. After incubation, the inoculated saliva was removed, and a growth medium comprising a mixture of 0.1 mL of 0.5% sucrose and 1.4 mL of basal medium mucin (BMM) was added. The 0.5% sucrose was prepared using saccharose (Daejung Chemicals & Metals Co., Ltd., Siheung, Republic of Korea). BMM was prepared by mixing 2.5 g/L of porcine mucin (Type II; Sigma Chemicals, St. Louis, MO, USA), 10.0 g/L of proteose peptone No. 3 (KisanBio, Seoul, Republic of Korea), 5.0 g/L trypticase peptone (KisanBio), 5.0 g/L yeast extract (KisanBio), 2.5 g/L potassium chloride (OCI Co., Ltd., Incheon, Republic of Korea), 0.06 g/L urea (Georgiachem, GA, USA), 0.1742 g/L arginine, 0.01 g/L menadione (Sigma Chemicals), and 0.05 g/L hemin (Sigma Chemicals), with the final pH adjusted to 7.0. Fresh growth medium was supplied every 24 h, and the oral microcosm biofilm was formed over 2 days.

### 2.5. Antibacterial Treatment

**Figure 2:**
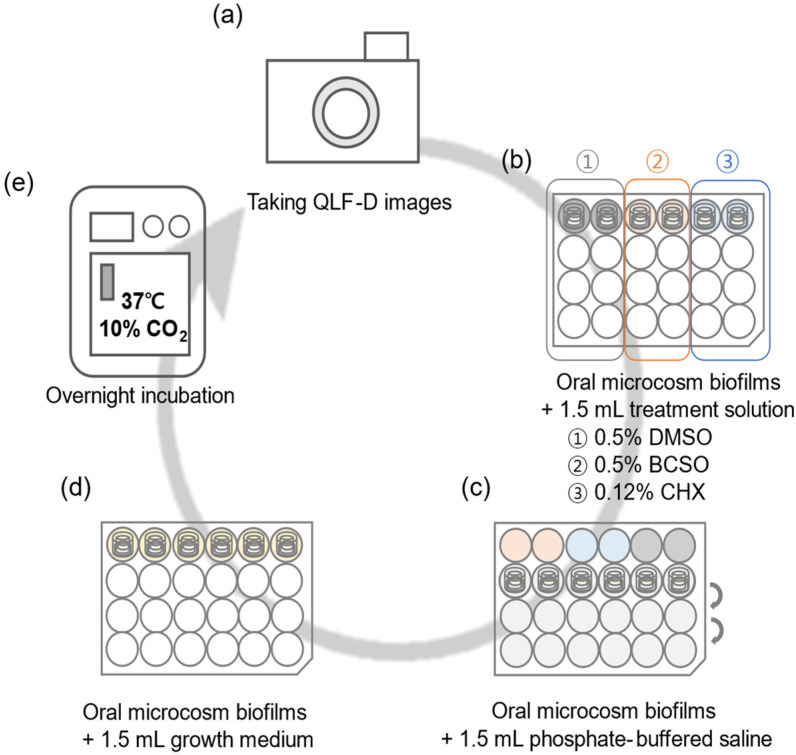
Flow diagram of oral microcosm biofilm treatment. (**a**) Before treatment, images of the oral microcosm biofilms were taken using a QLF-D camera. (**b**) Oral microcosm biofilms were immersed in treatment solution for 1 min. Three types of treatment solutions were tested (0.5% DMSO, 0.5% BCSO, and 0.12% CHX). (**c**) Oral microcosm biofilms were washed three times with phosphate-buffered saline. (**d**) Fresh growth medium (1.4 mL of basal medium mucin and 0.1 mL of 0.5% sucrose) was supplied to oral microcosm biofilms. (**e**) After treatment, the 24-well plate was placed in the incubator. BCSO, black cumin seed oil; CHX, chlorhexidine gluconate; DMSO, dimethyl sulfoxide; QLF-D, quantitative light-induced fluorescence-digital.

The formed oral microcosm biofilms were treated with 1.5 mL of 0.5% BCSO (experimental group), 0.5% DMSO (negative control group), or 0.12% CHX (hexamedine, Bukwang Pharm. Co., Ltd., Ansan, Republic of Korea; positive control group) for 1 min each. Following antibacterial treatment, the oral biofilms were rinsed three times with 1.5 mL of phosphate-buffered saline (PBS) for the removal of any residual solution. Fresh growth medium (1.5 mL) was then added, and the oral biofilms were incubated at 37 °C with 10% CO_2_. This treatment process was conducted at the same time each day and was repeated for a total of 6 days (Figure 2).

### 2.6. Evaluation of Red Fluorescence Intensity (Ratio_R/G_) of the Oral Microcosm Biofilm

**Figure 3:**
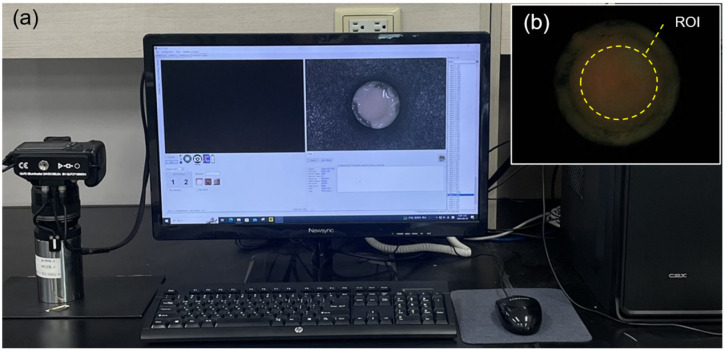
The quantitative light-induced fluorescence-digital (QLF-D) system. The images display (**a**) the QLF-D camera and C3 v1.16 software used for imaging and (**b**) the region of interest (ROI) for analysis of the red fluorescence intensity on the oral microcosm biofilm, calculated as the ratio of red pixels to green pixels in the fluorescence images using an image analysis program. ROI, region of interest.

Specimens were photographed daily using a quantitative light-induced fluorescence-digital (QLF-D) camera (QLF-D Biluminator™2+, Inspektor Research Systems BV, Amsterdam, the Netherlands) (Figure 3). The blue-light imaging conditions of the QLF-D camera were set to a shutter speed of 1/60 s, aperture value of 7.1, and ISO speed of 1600, whereas the white-light imaging conditions were set to a shutter speed of 1/60 s, aperture value of 8.0, and ISO speed of 250. The distance between the camera lens and the specimen was consistently maintained at 10 cm during imaging. The captured images were saved using C3 v1.16 software (Inspektor Research Systems BV) and analyzed using an image analysis program (Image PRO^®^ v. 10.0.8 Build 7088, 32-bit, Media Cybernetics, Inc., Silver Spring, MD, USA). The same region of interest (ROI) was set for all oral microcosm biofilm images. The red and green intensities within the ROI were measured, and the red fluorescence intensity was calculated as the ratio of red pixels to green pixels in the fluorescence images (Ratio_R/G_). A lower Ratio_R/G_ indicated lower pathogenicity of the oral biofilm.

### 2.7. Biomass Evaluation

In order to quantify the biomass, including both EPS and live bacterial cells, fluorescent staining was applied as follows. For fluorescent staining of EPS produced during oral microcosm biofilm formation, 1 nm of Alexa Fluor 647-dextran conjugate (647/668 nm; Thermo Fisher Scientific, Scoresby, Australia) was added to the growth medium, which was replaced daily. After antibacterial treatment for 6 days, the oral biofilms were rinsed three times with 1.5 mL of PBS for the removal of loosely attached microorganisms. The oral biofilm suspension was then prepared by vortexing and sonication for 1 min each. To stain the live bacteria within the oral biofilm, 1 μM of SYTO^®^ (480/500 nm, Thermo Fisher Scientific) was used for 15 min in the dark. After staining, 6 µL of the oral biofilm suspension was placed on a glass slide and covered with a coverslip. The prepared slides were observed at 10× magnification using a confocal laser scanning microscope (CLSM; LSM900, Carl Zeiss Microscopy, Jena, Germany) and imaging software (ZEISS ZEN Microscopy Software 3.6; Carl Zeiss). To quantify the red-stained EPS and green-stained live cells in each image, an image analysis program (Image PRO^®^ v. 10.0.8 Build 7088, 32-bit, Media Csybernetics, Inc., Rockville, MD, USA) was used to measure the red fluorescence intensity and green fluorescence intensity. Lower red and green fluorescence intensities mean less EPS produced during maturation and fewer live bacteria within oral biofilms, respectively.

### 2.8. Evaluation of Colony-Forming Units (CFUs) of Aciduric Bacteria

Brain heart infusion (BHI) agar plates were prepared by mixing 37 g of Bacto™ BHI (Becton Dickinson & Co., Franklin Lakes, NJ, USA) and 20 g of Bacto™ agar (Becton Dickinson & Co) in 1 L of distilled water, with the final pH adjusted to 4.8. On the final day of treatment, the oral microcosm biofilm suspension was prepared as previously described, and the suspension was serially diluted (from 10⁻^1^ to 10⁻^5^). Subsequently, 100 µL of each dilution was spread onto BHI agar plates. The plates were incubated at 37 °C with 10% CO_2_ for 72 h, following which, CFUs of aciduric bacteria were counted. The average CFU count/mL was calculated for each plate. A lower CFU count/mL indicated a lower survival rate for aciduric bacteria.

### 2.9. Statistical Analysis

All collected data were analyzed using SPSS Statistics ver. 28.0 (IBM Co., Armonk, NY, USA), with the level of significance set at 0.05. The normality of the data was assessed using the Kolmogorov–Smirnov test, and the homogeneity of variances was evaluated using Levene’s test. One-way ANOVA was performed to compare the effects between groups. Given that the assumption of homogeneity of variances was not always met, the Games–Howell post-hoc test, which is appropriate for data with unequal variances, was used.

## 3. Results

### 3.1. Red Fluorescence Intensity: Ratio_R/G_

**Figure 4:**
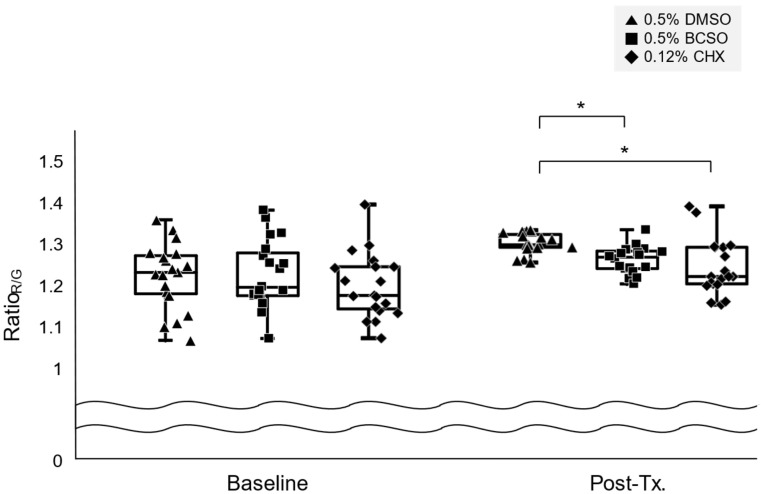
Changes in the Ratio_R/G_ of the oral microcosm biofilm following treatment. *: *p* < 0.05. The *p*-value was obtained using a one-way analysis of variance and the Games–Howell post-hoc test. Ratio_R/G_ refers to the ratio of red pixels to green pixels in fluorescence images of the oral microcosm biofilm, as captured by a quantitative light-induced fluorescence-digital camera. BCSO, black cumin seed oil; CHX, chlorhexidine gluconate; DMSO, dimethyl sulfoxide; Tx, treatment.

Ratio_R/G_ after treatment with 0.5% BCSO (1.26 ± 0.03) was not significantly different from that after treatment with 0.12% CHX (1.24 ± 0.07; *p* = 0.552, Figure 4). However, Ratio_R/G_ after treatment with 0.5% BCSO was 0.97 times lower than that after treatment with 0.5% DMSO (1.30 ± 0.02; *p* < 0.0001). 

**Figure 5:**
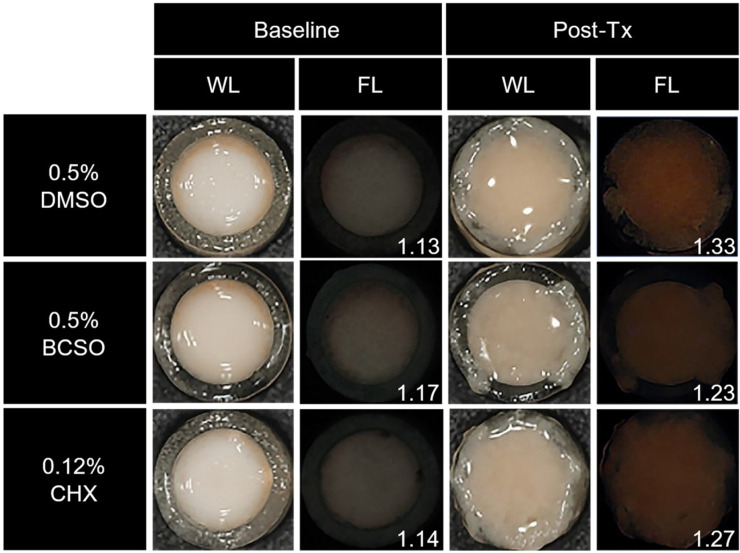
Representative images of oral microcosm biofilms captured using a quantitative light-induced fluorescence-digital (QLF-D) camera. The baseline images show the oral biofilm immediately before treatment, while the post-Tx images depict the oral biofilm after the sixth treatment. WL and FL images were captured using the white-light and blue-light sources of the QLF-D camera, respectively. The numbers at the bottom right indicate the red fluorescence intensity (Ratio_R/G_) of the oral biofilm. BCSO, black cumin seed oil; CHX, chlorhexidine gluconate; DMSO, dimethyl sulfoxide; FL, fluorescence image; Tx, treatment; WL, white-light image.

In the fluorescence images of the oral microcosm biofilm captured by the QLF-D camera, the oral biofilms in the 0.5% BCSO-treated and 0.12% CHX-treated groups exhibited weaker red fluorescence intensity than did the oral biofilms in the 0.5% DMSO-treated group (Figure 5).

### 3.2. Biomass

**Figure 6:**
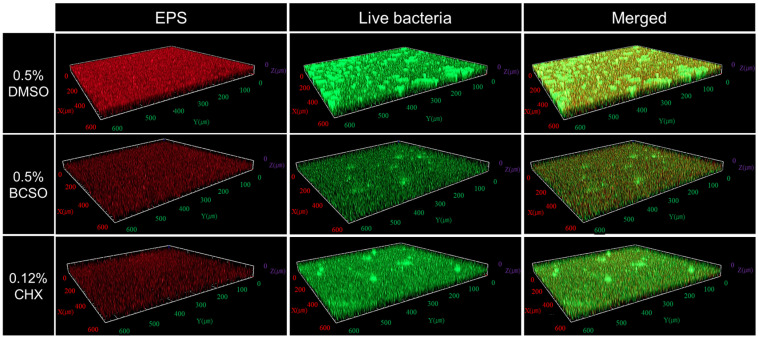
Representative confocal laser scanning micrographs (10× magnification) showing EPS and live bacteria within oral microcosm biofilms after treatment. The merged images are a combination of the EPS and live bacteria images displayed in the same row. BCSO, black cumin seed oil; CHX, chlorhexidine gluconate; DMSO, dimethyl sulfoxide; EPS, extracellular polymeric substance.

The biomass of the oral microcosm biofilm, as measured by evaluating the EPS and live bacteria counts using CLSM, was measured after treatment. Observation of the stained EPS in the oral biofilm using CLSM (Table 1, Figure 6) showed no significant difference between the 0.5% BCSO-treated and 0.12% CHX-treated groups (*p* = 0.743). However, the EPS levels after treatment with 0.5% BCSO were significantly lower than those after treatment with 0.5% DMSO by 0.38 times (*p* < 0.01).

The 0.5% BCSO-treated group had significantly fewer live bacteria, with a count that was 0.55 times lower than that in the 0.12% CHX-treated group and 0.30 times lower than that in the 0.5% DMSO-treated group (*p* = 0.018 and *p* < 0.001, respectively). 

### 3.3. CFUs of Aciduric Bacteria

After treatment with 0.5% BCSO, the CFUs of aciduric bacteria showed no significant difference from those after treatment with 0.12% CHX (*p* = 0.935, Table 2). However, the CFUs of aciduric bacteria were 0.96 times lower after treatment with 0.5% BCSO than after treatment with 0.5% DMSO (*p* < 0.05).

## 4. Discussion

This study compared the antibacterial effects of the natural extract BCSO with those of CHX on oral microcosm biofilms. The results led to the acceptance of our null hypothesis that there would be no significant difference between the antibacterial effects of BCSO and those of CHX on oral microcosm biofilms. 

Analysis of the red fluorescence intensity (Ratio_R/G_) in the oral microcosm biofilm revealed that the pathogenicity of the oral microcosm biofilm was suppressed to a similar extent by 0.5% BCSO and 0.12% CHX. The QLF-D camera, equipped with a special light source and filters, detects endogenous porphyrins, which are bacterial metabolites, and both exogenous and endogenous polysaccharides [25]. This detection allows for the degree of maturation and pathogenic potential of oral biofilms to be assessed. Previous studies have confirmed a strong correlation between the red fluorescence intensity and the maturity and cariogenicity of oral biofilms [26]. More mature and oral biofilms emit stronger red fluorescence [27]. This red fluorescence intensity is a useful indicator for evaluating and monitoring the effects of antibacterial agents on oral biofilms [28]. Therefore, our findings suggest that 0.5% BCSO and 0.12% CHX reduce the pathogenicity of oral biofilms by a comparable amount. Previous research has shown that BCSO exhibits similar or even greater growth-inhibitory effects on *S. mutans* biofilms than does CHX [13]. Microorganisms residing within actual biofilms possess resistance to antibacterial agents that is 1000 to 1500 times stronger than that shown by planktonic bacteria [29]. Despite this, 0.5% BCSO demonstrated effects similar to those of 0.12% CHX, even in the more resistant oral microcosm biofilm model used in this study. This finding suggests that BCSO is a potent natural antibacterial agent capable of maintaining its effectiveness in the complex, multi-species environment of actual oral biofilms.

Both 0.5% BCSO and 0.12% CHX reduced EPS to similar levels within the oral microcosm biofilm. EPS plays a crucial role in enhancing the mechanical stability of oral biofilms by promoting microbial adhesion to surfaces and cohesion between cells [30]. On the CLSM images, the 0.5% BCSO-treated group exhibited fewer EPS and a looser oral biofilm structure than did the 0.5% DMSO-treated group. After treatment with 0.5% DMSO, which was used as the solvent control, the amounts of EPS and live bacteria were higher than those of other agents. Previous studies have consistently shown that CHX significantly inhibits EPS production and bacterial adhesion, making it a potent agent in preventing biofilm development and dental caries [31]. These findings suggest that BCSO can effectively weaken the structural stability of oral biofilms by reducing EPS, thereby inhibiting the formation of pathogenic oral biofilms. Natural products can help in managing oral biofilms through their wide range of biological activities. Specifically, these extracts inhibit bacterial growth or acid production and interfere with glucosyltransferase activity, thereby preventing glucan synthesis in the EPS and blocking the adherence of bacterial cells to the tooth surface [32,33]. By inhibiting the formation of the EPS matrix, these natural extracts reduce biofilm stability and its ability to protect bacteria. Additionally, studies have shown that various extracts from *N. sativa* have potent inhibitory effects on oral biofilm formation [34]. Consistent with these findings, our results suggest that BCSO has the potential to act as an effective natural antibacterial agent by targeting EPS. Fewer live bacteria were present within the oral biofilm after treatment with 0.5% BCSO than after treatment with 0.12% CHX. This effect of BCSO may be attributed to its major active component, thymoquinone [19,35]. The exact mechanisms are still being studied; however, previous research has shown that thymoquinone inhibits the growth of specific pathogenic bacteria and can prevent the formation of pathogenic oral biofilms, which are the primary cause of bacterial diseases [36]. Additionally, studies on rats fed a diet containing thymoquinone demonstrated significantly lower levels of dental caries and plaque compared to control groups, suggesting its potent inhibitory effect on oral biofilm formation [37]. Thymoquinone reduces the secretion of virulence factors like pyocyanin and protease, which may contribute to its protective role in oral health by mitigating bacterial virulence and inhibiting biofilm formation [38].

Furthermore, no significant difference was observed in the CFUs of aciduric bacteria between the 0.5% BCSO-treated and 0.12% CHX-treated groups. This result suggests that 0.5% BCSO exhibits an effect similar to that of 0.12% CHX in reducing the survival of acidogenic, aciduric bacteria that contribute to caries. BCSO and CHX have different chemical compositions and mechanisms of action; however, both agents may target similar bacterial pathways or functions, such as cell membrane disruption or interference with aciduric bacterial metabolism. Previous studies have also confirmed that black cumin seeds possess strong antibacterial activity against oral pathogenic bacteria involved in dental caries, such as *S. mutans* and *S. sanguinis* [16,39]. These findings support our results, demonstrating the antibacterial effect of BCSO on aciduric bacteria.

This study had some limitations. Firstly, DMSO was used to dissolve non-polar BCSO; this solvent can cause side effects such as cytotoxicity, even at low concentrations. We used 0.5% DMSO in the control group to account for its potential effects; however, its inherent cytotoxicity could still have influenced the outcomes, particularly regarding bacterial viability and biofilm characteristics. Future studies should consider using alternative solvents with lower cytotoxicity or conducting additional controls to better isolate the effects of DMSO. Furthermore, evaluating the direct impact of DMSO on oral biofilm integrity and microbial activity would provide more clarity and allow for a more accurate interpretation of the results related to the antibacterial properties of BCSO. Secondly, the saliva used in the experiment was collected from a single donor. The microbial composition of saliva varies greatly between individuals [40]; therefore, collection of saliva from multiple donors could enhance the clinical diversity of oral microcosm biofilms [41]. Using saliva from a single donor may limit the microbial diversity and introduce bias by failing to represent the variety of oral environments in the general population. Future studies should aim to collect saliva from multiple donors to create more representative and clinically relevant biofilm models. This approach would allow for a broader assessment of the effects of antimicrobial agents and increase the generalizability of the findings. Thirdly, this study applied antibacterial treatment to oral biofilms that had matured for only 2 days. As oral biofilms mature, their biomass, including EPS levels, increases, which in turn increases the pathogenicity of these biofilms [42]. According to previous studies, oral biofilms that had matured for 6 days showed lower sensitivity to 0.12% CHX treatment than did those that had matured for only 2 days [43]. This finding suggests that the effectiveness of antibacterial agents may vary depending on the maturity of the oral biofilm, and our findings alone cannot generalize the effects of BCSO. Future in vitro studies should evaluate the effects of BCSO on oral biofilms in various stages of maturity in order to validate the clinical efficacy of BCSO. Future studies should assess the long-term efficacy of BCSO on more mature biofilms. This approach would provide valuable insights into the potential for BCSO to be used as an alternative to conventional treatments in various stages of biofilm development. Fourthly, the oral microcosm biofilm model used in this study has inherent limitations owing to the in vitro environment. The supply and flow of nutrients are restricted, and the absence of factors such as fluid dynamics and mechanical stimuli (such as chewing or brushing) leads to certain discrepancies compared with clinical conditions. Future studies could benefit from incorporating mechanical agitation to better mimic the natural oral environment, which would allow for a more accurate evaluation of the antimicrobial effects. Nevertheless, this model remains a valuable tool for studying antimicrobial agents owing to its simplicity, ease of use, and its ability to replicate the microbial communities found in the human oral cavity. Finally, in this study, we calculated the sample size using G*Power with an effect size of 0.43 to ensure statistical significance. However, given the complexity of the oral biofilm model and the potential for inter-individual variability, this effect size may not fully account for the differences in biofilm formation between individuals. A larger sample size or additional power analyses may be necessary in future studies to better capture these variations.

Despite these limitations, to the best of our knowledge, this study is the first to evaluate the effects of BCSO using the oral microcosm biofilm model, which most closely replicates the actual oral environment in an in vitro setting. Research on the effects of BCSO on oral health is extremely limited, and most previous studies have only assessed the antimicrobial effects of BCSO on single bacterial species. This study demonstrates that BCSO exhibits sufficient antibacterial effects on oral microcosm biofilms, comparable to those of existing chemotherapeutic agents like CHX, thus suggesting the potential of BCSO as an alternative treatment. Moreover, this study provides a foundation for future research to investigate the mechanisms of action, biocompatibility, and other aspects of BCSO, which are necessary for its commercialization. Further studies should consider various factors such as formulation, cost, and patient compliance with the drug to increase the clinical applicability of BCSO.

## 5. Conclusions

This study demonstrated that treatment with 0.5% BCSO reduced the pathogenicity, EPS levels, and aciduric bacteria in oral microcosm biofilms to levels similar to those achieved with 0.12% CHX, a conventional antibacterial agent. In addition, 0.5% BCSO demonstrated a greater antibacterial effect on live bacteria than 0.12% CHX. These findings represent an important first step in assessing the clinical potential of BCSO. Based on these results, we propose that BCSO, a naturally derived compound, could serve as a novel antibacterial agent with advantages such as fewer side effects and a reduced risk of bacterial resistance. Therefore, BCSO could replace conventional agents like CHX, which are associated with adverse effects.

## Figures and Tables

**Table 1:** Biovolume after antibacterial treatment.

Treatment	N	EPS	Live Bacteria
0.5% DMSO	10	16.23 ± 7.70 ^a^	28.15 ± 9.94 ^a^
0.5% BCSO	10	6.21 ± 1.65 ^b^	8.44 ± 2.72 ^b^
0.12% CHX	10	6.82 ± 1.97 ^b^	15.37 ± 6.21 ^c^
*p*-value		<0.001	<0.001

Data are presented as mean ± standard deviation. The *p*-value has been obtained using a one-way analysis of variance. ^a,b,c^ Different letters in the same column indicate significant differences between treatment groups according to the Games–Howell post-hoc test at α= 0.05. BCSO, black cumin seed oil; CHX, chlorhexidine gluconate; DMSO, dimethyl sulfoxide; EPS, extracellular polymeric substance.

**Table 2:** Mean CFUs of aciduric bacteria after antibacterial treatment.

Treatment	N	Log_10_CFUs/mL
0.5% DMSO	19	5.78 ± 0.22 ^a^
0.5% BCSO	19	5.56 ± 0.29 ^b^
0.12% CHX	19	5.52 ± 0.39 ^b^
*p*-value		0.028

Data are presented as mean ± standard deviation. The *p*-value is obtained using a one-way analysis of variance. ^a,b^ Different letters indicate significant differences between treatment groups according to the Games–Howell post-hoc test at α= 0.05. BCSO, black cumin seed oil; CFUs, colony-forming units; CHX, chlorhexidine gluconate; DMSO, dimethyl sulfoxide.

## Data Availability

The data presented in this study are available on request from the corresponding author. The data are not publicly available due to intellectual property rights restrictions.
